# CORDIC-Based General Multiple Fading Generator for Wireless Channel Digital Twin

**DOI:** 10.3390/s23052712

**Published:** 2023-03-01

**Authors:** Chen Fang, Kai Mao, Sheng Fang, Zikun Zhao, Boyu Hua, Tao Liu, Qiuming Zhu

**Affiliations:** 1 The Key Laboratory of Dynamic Cognitive System of Electromagnetic Spectrum Space, College of Electronic and Information Engineering, Nanjing University of Aeronautics and Astronautics, Nanjing 211106, China; 2 National Mobile Communications Research Laboratory, Southeast University, Nanjing 210096, China

**Keywords:** channel digital twin, channel fading generator, CORDIC, statistical properties

## Abstract

A wireless channel digital twin is a useful tool to evaluate the performance of a communication system at the physical or link level by generating the physical channel controllably. In this paper, a stochastic general fading channel model is proposed, which considered most of the channel fading types for various communication scenarios. By using the sum-of-frequency-modulation (SoFM) method, the phase discontinuity of the generated channel fading was well addressed. On this basis, a general and flexible generation architecture for channel fading was developed on a field programmable gate array (FPGA) platform. In this architecture, improved CORDIC-based hardware circuits for the trigonometric function, exponential function, and natural logarithm were designed and implemented, which improved the real-time performance of the system and the utilization rate of the hardware resources compared with the traditional LUT and CORDIC method. For a 16-bit fixed-point data bit width single-channel emulation, the hardware resource consumption was significantly reduced from 36.56% to 15.62% for the overall system by utilizing the compact time-division (TD) structure. Moreover, the classical CORDIC method brought an extra latency of 16 system clock cycles, while the latency caused by the improved CORDIC method was decreased by 62.5%. Finally, a generation scheme of a correlated Gaussian sequence was developed to introduce a controllable arbitrary space–time correlation for the channel generator with multiple channels. The output results of the developed generator were consistent with the theoretical results, which verified the correctness of both the generation method and hardware implementation. The proposed channel fading generator can be applied for the emulation of large-scale multiple-input, multiple-output (MIMO) channels under various dynamic communication scenarios.

## 1. Introduction

Channel fading plays an important role for the realistic performance assessment of communication technologies [1]. The digital twin is becoming a key technology to optimize wireless communication algorithms and evaluate the performance of wireless communication systems [2,3,4]. General channel fading reproduction (or a digital twin) for arbitrary communication scenarios in the laboratory is an effective way to speed up the validation, evaluation, and optimization of new communication technologies at the physical or link level [5,6,7]. However, the existing channel emulator architecture based on the stochastic channel model has poor universality and is only suitable for the emulation of channel characteristics for several specific communication scenarios [8,9]. In addition, the traditional channel fading emulation methods are limited by the utilization rate of field programmable gate array (FPGA) hardware resources and the real-time performance of the algorithm when they are applied for larger-scale multiple-output, multiple-output (MIMO) channels or highly dynamic channels in B5G/6G [10,11].

There are various scenario-dependent channel fading models reported in the literature [12,13,14,15,16]. For example, by analyzing massive measurement data, channel fading was described as a Nakagami distribution in the suburban scenario [12]. Due to the blocking by buildings and mountains, it was modeled in [13] as a lognormal distribution in a built-up area. For urban areas, the Weibull distribution was adopted in [14] to evaluate the network-level performance. Moreover, the compound fading types such as shadowed-Rician are suitable for the channel fading emulation of satellite and terrestrial links [15,16]. The authors in [17,18,19] proposed software-based simulators to generate the channel fading such as Corazza, Loo, and NLN. However, with the increase of the computational complexity, these software-based methods’ results have poor real-time performance.

The traditional sum-of-sinusoids (SOS) method and its derivations were developed for real-time hardware implementation [14,20,21,22,23]. However, the phase of output fading is not continuous and smooth due to the idea of the piecewise operation in the SoS method. Li and Zhu proposed the sum-of-frequency-modulation (SoFM) method, which can ensure the continuity of the Doppler phase [20]. Several representative hardware generators based on an FPGA platform were addressed in [14,20,21,22,24,25]. Among them, the most popular one is based on the cosine lookup table (LUT) method [20,22]. The simple hardware structure of this method can generate various nonlinear functions. However, a huge amount of random-access memory (RAM) resource is needed for a higher emulation accuracy and a larger MIMO scale. The CORDIC algorithm is another low-cost hardware implementation method, which can realize the fixed-point calculation of various elementary functions and only consumes a small amount of RAM resource compared to the LUT method. The authors in [14] developed a Weibull and Suzuki fading generator based on the traditional CORDIC method, but it caused a long initialization latency of 16 system clock cycles due to the large number of iterations. To reduce the iteration number and time delay, the authors in [26] improved the CORDIC algorithm by using the pipeline architecture of an FPGA and reduced the system delay to 12 clock cycles. However, this method took a large update time for the channel parameters, which is not suitable for non-stationary channel generation. The authors in [21] introduced an approximated rotation factor state, and the authors in [24,25] proposed a high radix algorithm that can further reduce the iteration latency of the CORDIC algorithm. However, the hardware circuit was complex and difficult to implement. Therefore, considering the complexity of the hardware implementation and the update cycle of time-varying channel parameters, this paper proposes an improved CORDIC method to emulate arbitrary channel fading. This paper aimed to (1) fill the above research gap and develop an efficient and universal digital twin method for arbitrary channel fading, (2) propose a correlated multi-Gaussian sequence generation architecture, and (3) achieve a controllable space–time correlation for MIMO channels. Overall, the major contributions and novelties are summarized as follows:

(1) A novel FPGA-based hardware generation architecture for general channel fading in various communication scenarios was developed. The new architecture takes most of the channel fading into account, i.e., Rayleigh, Rice, Weibull, Nakagami, lognormal, Suzuki, Corazza, NLN, and Loo, and can be applied to rural, urban, suburban, and high-speed rail scenarios, especially land mobile satellite communication scenarios. Moreover, by updating the hardware parameters in real-time, the proposed architecture also supports the non-stationary channel fading under dynamic scattering scenarios with a continuous channel phase. It can be used to map the physical channel to the controllable and repeatable digital space.

(2) The CORDIC-based hardware circuits for the trigonometric functions, the exponential function, and the natural logarithm were developed, which consisted of an angle mapping module, angle recording module, and iteration module. By using a greedy CORDIC parallel pipeline structure and domain folding technique, the proposed method consumes less RAM resource and has better real-time performance than existing methods. The inherent delay caused by the improved CORDIC method of channel fading emulation is less than the parameter updating period, which is more suitable for the emulation of non-stationary channel fading. Moreover, the arbitrary space–time correlation of output channel fading can be controlled by choosing proper parameters based on the improved hardware implementation architecture. It is beneficial for the spatial–temporal correlated channel fading generation, i.e., MIMO channel and diversity channel.

(3) The proposed generator was implemented on a Xilinx XC7K325TFFG900 FPGA chip. The hardware emulation results showed that, compared with the traditional LUT method, the hardware resource of the improved CORDIC method was reduced from 9.14% to 6.21% when a single Gaussian sequence was generated in single-channel fading. Especially, with the increasing scale of MIMO, the number of channels to be emulated increased. The reduction of the hardware resource consumption was more significant when using our proposed greedy CORDIC method. In addition, the latency was cut by 62.5% compared to the classical CORDIC method, which is vital to support non-stationary channel emulation with fast time-variant channel characteristics. The output PDF agreed well with the theoretical one, and the average deviation was below 1.63% for a 16-bit data width. Moreover, the mean absolute error was merely 0.46% for CCF, and the average deviation was about 5.62% for ACF, which verified the correctness of the designed channel emulator.

The remainder of this paper is organized as follows. In Section 2, a channel model with general fading under different scenarios is illustrated. Section 3 gives the hardware architecture and FPGA implementation. A CORDIC-based generation method is developed for generating arbitrary channel fading. Meanwhile, a correlated multiple Gaussian sequence generation scheme is also given to introduce the auto- and cross-correlations between different channel fading types. Section 4 compares the hardware resource consumption and validates the generated channel fading. Finally, some conclusions are given in Section 5.

## 2. General Fading Channel Model

Based on radio propagation theory, the received signal consists of multiple paths with different delays and angles due to reflection, refraction, and diffraction. The channel impulse response (CIR) can be modeled as

(1) $$h\left(t,\tau \right)\;=\sum_{m=1}^{M}\{\sqrt{P_{m}\left(t\right)}\cdot r_{m}^{i}\left(t\right)\cdot e^{j\phi_{m}\left(t\right)}\cdot \delta \left(\tau -\tau_{m}\right)\}+n\left(t\right)\;$$

where *M* is the number of valid path, n(t) is the additive channel noise, and $P_{m}\left(t\right)$, $r_{m}^{i}\left(t\right)$, $\phi_{m}\left(t\right)$, and $\tau_{m}$ represent the path loss, the amplitude of channel fading such as small-scale fading and large-scale fading (or shadowing), the phase, and the delay of the *m*th path, respectively; i=RL,R,Na,Log,S,W,Loo,Co,NLN denote different types of channel fading.

Note that the channel fading in (1) can be modeled and generated in a deterministic or stochastic way. For the deterministic model, the fading parameters can be obtained by using the ray tracing (RT) method on specific communication scenarios. However, for some applications, we may have no detailed geometry information and the stochastic models based on measured data would be used. Table 1 gives widespread stochastic models of channel fading, which cover most of the communication scenarios. Deeply understanding and precisely reproducing these channel fading types are very useful for the exploration, design, and optimization of the communication systems.

## 3. Channel Fading Generation and Implementation

### 3.1. Overview of Hardware Generation Architecture

Due to the unique characteristics of the pipeline hardware circuit structure and parallel operation, FPGAs can reduce the complexity of hardware operation, improve the efficiency, and reduce the operation time, so they are suitable for the hardware emulation of complicated and time-variant channel fading. Considering that the channel model in (1) cannot be directly implemented in an FPGA, we firstly transferred it to the discrete model before the hardware implementation. The complex CIR can be rewritten in the discrete domain as

(2) $$\begin{array}{c} h\left(l,\xi \right)\;=\sum_{m=1}^{M(l)}\{\sqrt{P_{m}\left(l\right)}\cdot r_{m}^{i}\left(l\right)\cdot e^{j\phi_{m}\left(l\right)}\cdot \delta \left(\xi -{\left\lfloor \tau_{m}\left(l\right)\right\rfloor }_{Ts}\right)\}+n\left(l\right) \end{array}$$

where *l* and ξ are the discrete indexes in the time and delay domains, respectively, and ${\left\lfloor \tau_{m}\left(l\right)\right\rfloor }_{Ts}$ denotes the discrete path delay. Note that the channel fading factor $r_{m}^{i}\left(t\right)$ in (2) is the key to reproducing different channel models. Therefore, we mainly focused on the generation of $r_{m}^{i}\left(t\right)$ in this paper [28,29,30].

The Gaussian sequence is a discrete random sequence, and all elements are Gaussian variables. Let us define the *m*th discrete Gaussian sequence $u_{m}\left(l\right)$∼$N(0,\sigma_{m}^{2})$ with the variance $\sigma_{m}^{2}$; the Rayleigh fading can be expressed as

(3) $$\begin{array}{c} r_{m}^{\mathrm{RL}}\left(l\right)=\left|u_{m}\left(l\right)+j\cdot {\hat{u}}_{m}\left(l\right)\right| \end{array}$$

where *l* is the discrete sampling time and $u_{m}\left(l\right),{\hat{u}}_{m}\left(l\right)$ represent the Gaussian sequence and its Hilbert transformation, respectively; both are independent orthogonal components of Rayleigh fading. Similarly, Rice, Weibull, and Nakagami fading can be expressed as

(4) $$\begin{array}{c} \;\;r_{m}^{R}\left(l\right)=\left|\rho_{m}\cdot \exp\left(j\left(2\pi f_{m,\rho }l+\;\theta_{m,\rho }\right)\right)+\;u_{m}\left(l\right)+j\cdot {\hat{u}}_{m}\left(l\right)\right|\;\; \end{array}$$

(5) rmW(l)=exp(wmwm22·ln(rmRL(l)))

(6) $$\begin{array}{c} r_{m}^{\mathrm{Na}}\left(l\right)=\sqrt{u_{m,1}^{2}\left(l\right)+u_{m,2}^{2}\left(l\right)+\cdots +u_{m,2q}^{2}\left(l\right)} \end{array}$$

where $\rho_{m}$, $f_{m,\rho }$, and $\theta_{m,\rho }$ are the amplitude, Doppler frequency, and phase of the line-of-sight path of Rice fading, respectively, $w_{m}$ is the shape factor of Weibull fading, and 2*q* denotes the number of independent Gaussian sequences of Nakagami fading.

Furthermore, the lognormal fading can be expressed as

(7) $$\begin{array}{c} r_{m}^{\mathrm{Log}}\left(l\right)=\exp\left(\sigma_{m}^{\mathrm{Log}}u_{m}\left(l\right)+\mu_{m}^{\mathrm{Log}}\right) \end{array}$$

where $\sigma_{m}^{\mathrm{Log}}$ and $\mu_{m}^{\mathrm{Log}}$ are the standard deviation and mean value of lognormal fading, respectively. Finally, Suziki, Corazza, NLN, and Loo fading can be obtained by the combination of (2)–(6) as

(8) $$\begin{array}{c} r_{m}^{S}\left(l\right)=r_{m}^{\mathrm{RL}}\left(l\right)r_{m}^{\mathrm{Log}}\left(l\right) \end{array}$$

(9) $$\begin{array}{c} r_{m}^{Co}\left(l\right)=r_{m}^{R}\left(l\right)r_{m}^{\mathrm{Log}}\left(l\right) \end{array}$$

(10) $$\begin{array}{c} r_{m}^{\mathrm{NLN}}\left(l\right)=r_{m}^{\mathrm{Na}}\left(l\right)r_{m}^{\mathrm{Log}}\left(l\right) \end{array}$$

(11) $$\begin{array}{c} \begin{array}{c} \begin{array}{c} \begin{array}{c} r_{m}^{\mathrm{Loo}}\left(l\right)=|r_{m}^{\mathrm{Log}}\left(l\right)\rho_{m}\cdot \exp(j\left(2\pi f_{m,\rho }l+\theta_{m,\rho }\right)\\ +u_{m}\left(l\right)+j\cdot {\hat{u}}_{m}\left(l\right)| \end{array} \end{array} \end{array} \end{array}$$

**Figure 1:**
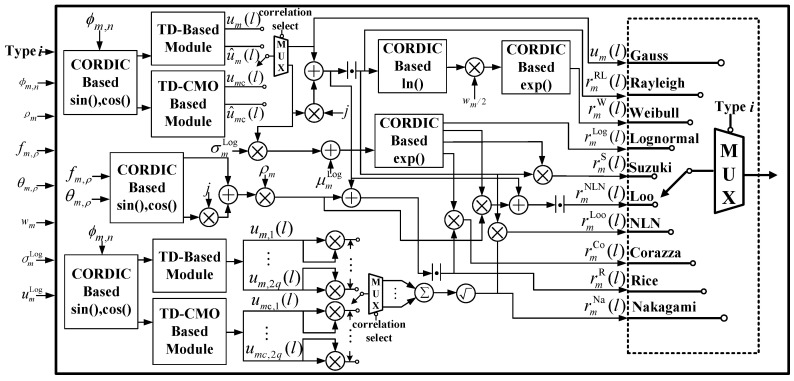
Hardware structure of general channel fading generation.

The Hardware structure of general channel fading generation is shown in Figure 1. Based on (2)–(10), the $\phi_{m,n}$ of the *n*th ray within the *m*th valid path, the amplitude $\rho_{m}$, frequency $f_{m,\rho }$, initial phase $\theta_{m,\rho }$ of the line-of-sight path, and other controlling factors $\sigma_{m}^{\mathrm{Log}}$, $\mu_{m}^{\mathrm{Log}}$, and $w_{m}$ can be configured by the user via a periphery interface. Note that the system only consists of several multipliers, accumulators, adders, and key modules for specific functions, i.e., trigonometric function, exponential function, natural logarithm function, and correlated multiple Gaussian sequences generation, which is used to implement the correlation matrix operation (CMO). The final output is controlled by a multiplexer according to the user-defined fading type.

### 3.2. CORDIC-Based Trigonometric Operation

The Gaussian sequence is the key to the proposed hardware architecture, so it is essential to develop an efficient hardware algorithm to generate multiple Gaussian variables. Based on the central limit theorem, a Gaussian random sequence can be modeled as the superposition of several cosine waves with random initial phases and different frequencies. Different from the traditional pseudo-random-bit-based generator, it can control the spectrum shape by choosing proper phase and frequency parameters [23]. Thus, an efficient trigonometric operation for cosine calculation is needed. So far, the LUT is the most-widespread method due to its good real-time performance, but it consumes much memory resource. For the B5G/6G communication system evaluation, the channel emulator needs to emulate non-stationary channel scenarios, where the channel parameters change dynamically in real-time [7]. The CORDIC module is initialized after the parameters are updated, which would cause latency for the valid channel fading output every time. Therefore, the system latency should be reduced as much as possible in emulating fast time-variant non-stationary channels. The traditional CORDIC algorithm consumes less hardware resource, but has the shortcoming of high latency with a 16-system-clock-cycle delay.

**Figure 2:**
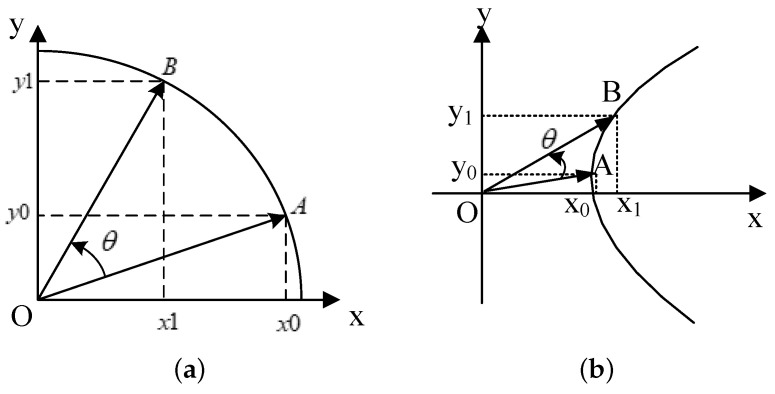
(**a**) Circular and (**b**) hyperbolic rotation model.

The basic idea of CORDIC is to decompose the trigonometric operation into a set of linear combinations of micro-rotation angles, as shown in Figure 2. A two-dimensional vector $\left(x_{0},y_{0}\right)$ is rotated by an angle θ to calculate the rotated vector $\left(x_{1},y_{1}\right)$ under the two rotation models. This transformation can be expressed, respectively, as [31]

(12) $$\begin{array}{c} \left[\begin{array}{c} x_{1}\\ y_{1} \end{array}\right]=\left[\begin{array}{cc} \cos\theta & -\sin\theta \\ \sin\theta & \cos\theta \end{array}\right]\left[\begin{array}{c} x_{0}\\ y_{0} \end{array}\right] \end{array}$$

(13) $$\begin{array}{c} \left[\begin{array}{c} x_{1}\\ y_{1} \end{array}\right]=\left[\begin{array}{cc} \cosh\theta & -\sinh\theta \\ \sinh\theta & \cosh\theta \end{array}\right]\left[\begin{array}{c} x_{0}\\ y_{0} \end{array}\right] \end{array}$$

Using the traditional CORDIC method to generate the trigonometric operation requires 16 iterative operations, so it will bring 16 clock cycles of latency [24], which is unacceptable for the real-time performance of the system. To tackle the issue of the high latency due to multiple iterations, a greedy CORDIC-based trigonometric operation is developed in this section. The target angle θ is encoded as a linear combination of the micro-rotation angle set denoted by {0≤k≤L−1|ϑ(k)}. The proposed method contains three main parts, i.e., angle mapping module, angle recording module, and iteration module.

**Figure 3:**
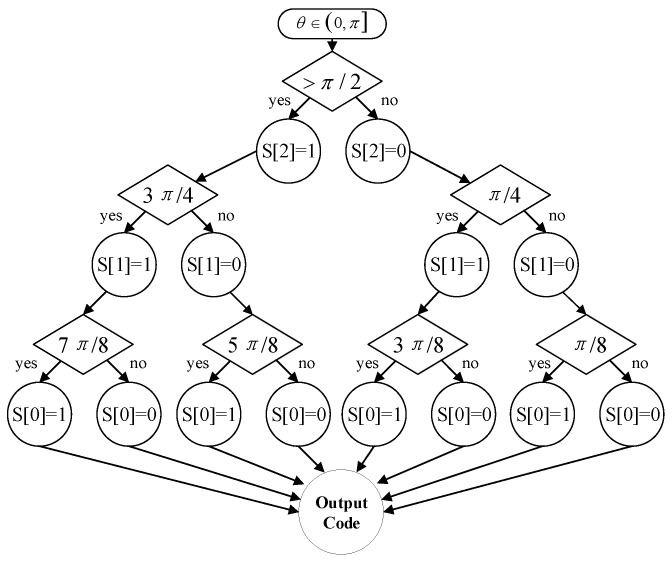
Angle mapping via binary coding.

The angle mapping module aims to map the input target phase $\phi_{m,n}\in \left(0,2\pi \right]$ to a smaller angle range. It yields a significant advantage of computational complexity when narrowing the angle range. In this paper, we divided the input angle into 16 equal domains with the uniform span of π/8. Let us take the first and second quadrants as an example: angle mapping can be realized through binary coding based on the domain folding, as shown in Figure 3.

**Figure 4:**
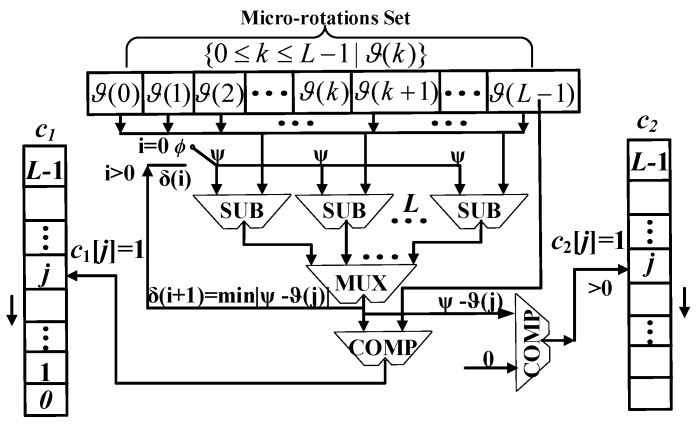
Greedy-based angle recording module.

The original input angle phase can be mapped to ϕ∈[0,π/8] by the angle mapping, then we can reduce the iteration number by reducing the redundant computation of unnecessary micro-rotation angles in the iteration process. The greedy-based angle recording module is shown in Figure 4. Based on the set of micro-rotation angles {0≤k≤L−1|ϑ(k)}, we used *L* subtractors and one multiplexer to express the mapped angle ϕ with several micro-rotation angles and *L*-bit codes $c_{1},c_{2}$, where $c_{1},c_{2}$ are used to compute the scaling factor, the direction of the sign function, and the shift value in the iteration unit. This process adopts a parallel pipeline structure to ensure $c_{1},c_{2}$ are generated within one clock cycle. Since the magnitude of the sign function is 1 and the sequence ϑ(i) is decreasing, the convergence of this process can be proven.

**Figure 5:**
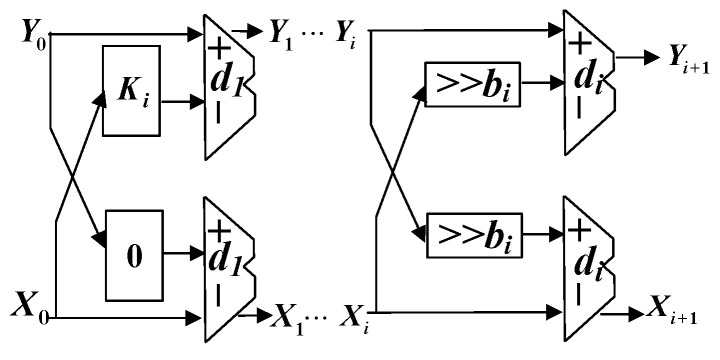
CORDIC-based circuit of the trigonometric function.

By factoring cosθ and the approximation equation $\tan\left(\vartheta \left(k\right)\right)\approx 2^{-k}$, the calculation can be simplified by several shifts and add operations. Then, we can obtain the direction of sign function $d_{i}$ and shift value $b_{i}$ by determining the value of each bit within $c_{1},c_{2}$ in the *i*th iteration process. Figure 5 shows one stage of the iterative structure, and it can be described as

(14) $$\begin{array}{c} \begin{array}{c} x_{{}^{i+1}}=\left(x_{i}-d_{i}\cdot y_{i}\cdot 2^{-b_{i}}\right)\\ y_{{}^{i+1}}=\left(y_{i}+d_{i}\cdot x_{i}\cdot 2^{-b_{i}}\right) \end{array} \end{array}$$

To calculate the trigonometric function, the values of $x_{0}$ and $y_{0}$ were initialized, respectively, to *K* and 0. To maintain the accuracy and avoid overflow, the scaling factor *K* can be further expressed as

(15) K=∏c1(j)≠0cosϑ(j)=∏c1(j)≠0111+2−2j1+2−2j.

By combining (11) and (13), it can be proven that the final output after several iterations can be expressed as

(16) $$\begin{array}{c} \left[\begin{array}{c} x_{n}\\ y_{n} \end{array}\right]\;\;=\;\;\frac{1}{x_{0}}\;\;\left[\begin{array}{cc} \cos\phi & -\sin\phi \\ \sin\phi & \cos\phi \end{array}\right]\;\;\left[\begin{array}{c} x_{0}\\ y_{0} \end{array}\right]\;\;=\;\;\left[\begin{array}{c} \cos\phi \\ \sin\phi \end{array}\right] \end{array}$$

After several iterations, the output values of $x_{n}$ and $y_{n}$ are obtained to be cosϕ and sinϕ, respectively. By combing the mapping relationship in the angle mapping module, we can recover the trigonometric function value of $\phi_{m,n}$.

### 3.3. CORDIC-Based Exponential and Natural Logarithm Operation

In this paper, the CORDIC-based exponential operation was implemented in the rotation mode of (12). The input phase for computing the exponential value was obtained by decomposing the rotation through a set of micro-rotation angles denoted by $\left\{1\leq k\leq L-1|\vartheta \left(k\right)=\tanh^{-1}\left(2^{-k}\right)\right\}$. Similarly, by factoring coshθ and $\tanh\left(\vartheta \left(k\right)\right)\approx 2^{-k}$, the iteration process can be expressed as

(17) $$\begin{array}{c} \begin{array}{c} \left[\begin{array}{c} x_{n}\\ y_{n} \end{array}\right]=\cosh\theta \left[\begin{array}{cc} 1 & \tanh\theta \\ \tanh\theta & 1 \end{array}\right]\left[\begin{array}{c} x_{0}\\ y_{0} \end{array}\right]\\ =\prod_{i=1}^{n}\left[\begin{array}{cc} 1 & d_{i}2^{-i}\\ d_{i}2^{-i} & 1 \end{array}\right]\left[\begin{array}{c} x_{0}\\ y_{0} \end{array}\right]K_{e} \end{array} \end{array}$$

where $d_{i}$ denotes the direction of the sign function with value ±1, and $K_{e}$ represents the scaling factor and can be further expressed as

(18) Ke=∏i=1ncoshϑ(i)=∏i=1n11(1−tanh2ϑ(i))(1−tanh2ϑ(i))=∏i=1n11(1−2−2i)(1−2−2i)

For the hyperbolic CORDIC, it should be noted that, when the iterative sequence number equals $k_{n}\left(k_{n}=3k_{n-1}+1,k_{1}=4,n\in Z^{+}\right)$, the corresponding stage of iterations should be executed again to ensure the algorithm converges [31]. The iterative recursive equation can be derived as

(19) $$\begin{array}{c} \begin{array}{c} x_{n+1}=x_{n}+d_{n}\cdot y_{n}\cdot 2^{-n}\\ y_{n+1}=y_{n}+d_{n}\cdot x_{n}\cdot 2^{-n}\\ z_{n+1}=z_{n}-d_{n}\cdot \vartheta \left(n\right) \end{array} \end{array}$$

To calculate the exponential function, the values of $x_{0}$, $y_{0}$, and $z_{0}$ are initialized, respectively. Let us take the lognormal fading as an example: we set $x_{0}\;=\;1$, $y_{0}\;=\;0$, and $z_{0}\;=\;\sigma_{m}^{\mathrm{Log}}u_{m}\left(l\right)+\mu_{m}^{\mathrm{Log}}$, respectively. After several iterations, when $z_{n}$ equals 0, the output values of $x_{n}$ and $y_{n}$ can be obtained as $\cosh(\sigma_{m}^{\mathrm{Log}}u_{m}\left(t\right)+\mu_{m}^{\mathrm{Log}})$ and $\sinh(\sigma_{m}^{\mathrm{Log}}u_{m}\left(t\right)+\mu_{m}^{\mathrm{Log}})$. Then, the output of $r_{m}^{\mathrm{Lo}}\left(t\right)$ can be obtained by the sum of $x_{n}$ and $y_{n}$.

The natural logarithm operation has a similar architecture as the exponential operation, except the operation of the mode. In the vectoring mode, the direction of $d_{n}$ is determined by the sign function of $y_{n}$. Based on the equation of ln(θ)=2tanh−1(θ−1θ−1θ+1θ+1), the natural logarithm function can be calculated by setting different initial values, i.e., $x_{0}\;=\;\theta +1$, $y_{0}\;=\;\theta -1$, and $z_{0}\;=\;0$. Taking Weibull fading as an example, the initial values were set as $x_{0}\;=\;\left|r_{m}^{RL}\left(t\right)\right|+1$, $y_{0}\;=\;\left|r_{m}^{RL}\left(t\right)\right|-1$, and $z_{0}=0$, respectively. After several iterations, when $y_{n}$ equals 0, the output is zn=tanh−1((rmRL(t)−1)(rmRL(t)−1)(rmRL(t)+1(rmRL(t)+1)) and $r_{m}^{W}\left(l\right)$ can be obtained by the definition in Table 1.

**Figure 6:**
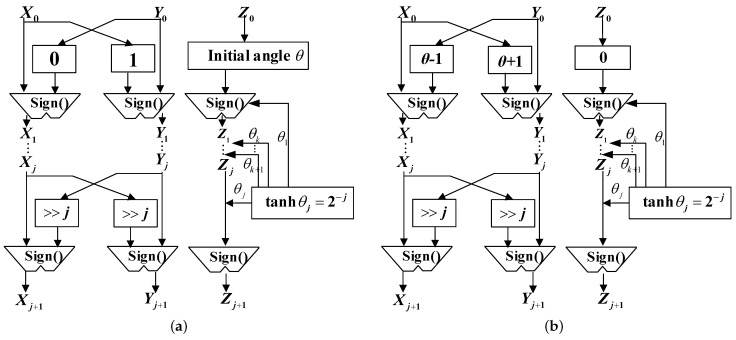
CORDIC-based circuits of (**a**) exponential and (**b**) natural logarithm operations.

Figure 6 gives one stage of the CORDIC-based structure for the exponential and natural logarithm operations. The sign function is determined by the remaining angle $Z_{j}$ in each iteration. The shift value is also determined by the iterative sequence number in each iteration. Note that the architecture uses a parallel pipeline structure to ensure that all the variables of each stage can be generated within a clock cycle.

### 3.4. Correlated Multiple Gaussian Sequence Generation

**Figure 7:**
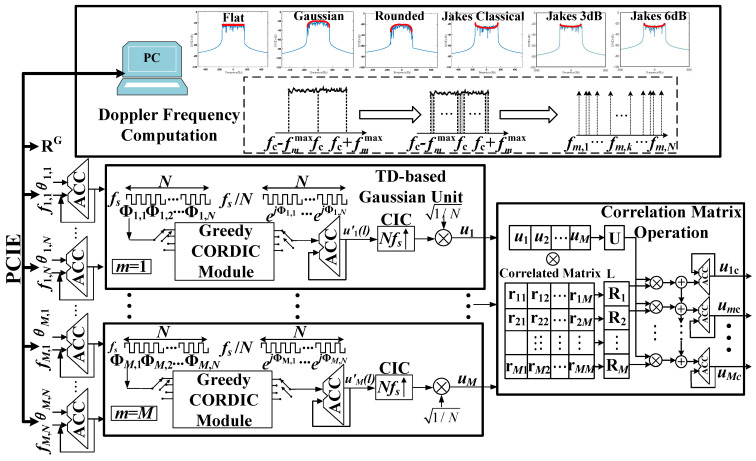
Overview of correlated multiple Gaussian sequence generation.

The temporal autocorrelation and spatial cross-correlation of multiple channels are very important, especially for the antenna arrangement, placement, and evaluation of MIMO systems [32]. To control the correlation of different channels or paths, we developed a correlated multiple Gaussian sequence generation scheme, as shown in Figure 7. It consists of three parts, i.e., the Doppler frequency computation, a time-division (TD)-based Gaussian unit, and the correlation matrix operation.

The SoS or sum-of-cisoids (SoC) method is widely adopted to generate channel fading under stationary scenarios. In order to take the non-stationary aspects into account, the dynamic update of the channel parameters is necessary for the non-stationary channel emulation under highly mobile scenarios such as high-speed trains and UAV communications. Different from the method of a non-stationary channel, it is emulated by a traditional segmented stationary channel, which updates the channel parameters at each stationary channel stage [33,34]. However, the continuity of the channel phase will not be guaranteed by this method. In order to precisely reproduce the non-stationary aspects, it is important to confirm the continuity of the output channel fading between adjacent channel states. Therefore, the channel parameters are updated in the discrete domain by the SOFM method as [20,35]

(20) $$\begin{array}{c} u_{m}\left(l\right)=\sum_{n=1}^{N}\frac{1}{\sqrt{N}}e^{\left(2\pi \sum_{k=0}^{l}T_{s}f_{m,n}\left[k\right]+\theta_{m,n}\right)} \end{array}$$

where *N* is the number of branches and $f_{m,n}$ and $\theta_{m,n}$ are the discrete Doppler frequency and initial phase uniformly distributed over $\left(0,2\pi \right]$, respectively.

Note that the discrete Doppler frequency in (19) controls the autocorrelation of the generated sequence. and it is determined by user-defined information, i.e., the DPSD power spectral density $S_{\mu_{m}\mu_{m}}\left(f\right)$, the carrier frequency $f_{c}$, and the maximum Doppler frequency $f_{m}^{\max}$. We firstly sampled the continuous input DPSD with a short time interval. The Doppler frequencies $f_{1}$, $f_{2}\cdots f_{N}$ were calculated by the MEA method, which makes the area under the DPSD curve within the range $\left[-f_{m}^{\max},f_{m}^{\max}\right)$ equal to $\sigma_{m,u}^{2}/N$.

(21) $$\begin{array}{c} \int_{-f_{m}^{\max}}^{f_{m}^{\max}}S_{\mu_{m}\mu_{m}}\left(f\right)df=\frac{\sigma_{m,u}^{2}}{N},n=1,2,\ldots ,N \end{array}$$

The Gaussian generation unit based on the greedy-based CORDIC module is shown in Figure 7. In order to reduce the hardware resource consumption due to massive branches, it was implemented using a TD scheme. The input phase of *N* rays is obtained by the Doppler frequency and initial phase, which are loaded via a high-speed transmission interface such as the PCIE bus. For each valid path, all *N* branches are calculated by the greedy CORDIC module and summed up by an ACC serially. The data rate of ACC output $u_{m}^{\prime }\left(l\right)$ is reduced to one over *N* of the initial rate. To match the data rate, $u_{m}^{}\left(l\right)$ is interpolated *N*-times with a cascade integrator comb (CIC) filter. Similarly, this TD scheme can be applied to *M* valid paths through a multiplexer.

On the other hand, a correlation matrix $R^{G}$ is introduced to control the cross-correlation between different Gaussian sequences, which can also improve the performance of ergodicity and orthogonality. The conventional correlated matrix $R^{G}$ is decomposed into a low triangular matrix *L* by the Cholesky decomposition. We found that it only works for the positive correlation matrix. To overcome this shortcoming, the eigenvalue decomposition method was adopted in this paper as

(22) $$\begin{array}{c} \begin{array}{c} R^{G}\;\;\;=\;\;\;V\bar{\Lambda }\bar{\Lambda }V^{H},\Lambda \;=\;diag\left(\lambda_{1},\lambda_{2},\cdots \lambda_{M}\right)\\ \bar{\Lambda }\;\;\;=\;\;\;\sqrt{diag({\hat{\lambda }}_{1},{\hat{\lambda }}_{2},\cdots ,{\hat{\lambda }}_{M})},{\hat{\lambda }}_{j}\;=\;\left\{\;\;\begin{array}{c} \lambda_{j}\begin{array}{cc} & \lambda_{j}\geq 0 \end{array}\\ 0\begin{array}{cc} & \lambda_{j}<0 \end{array} \end{array}\right. \end{array} \end{array}$$

where $\lambda_{j}$ is the eigenvalue of matrix $R^{G}$. Then, the decomposed matrix $L=V\bar{\Lambda }$ is multiplied by the spatial independent Gaussian sequences. The cross-correlated Gaussian sequences can be obtained by $U=\left[u_{1c}u_{2c}\cdots u_{Mc}\right]$ and *L*.

To illustrate the above generation method, we took four temporally and spatially correlated Gaussian sequences denoted by $h_{11},h_{12},h_{21},h_{22}$ as an example. The DPSDs of the four sequences were assumed to be the same as the classical Jakes shape. The desired cross-correlation matrix $R^{G}$ is given as

(23) $$\begin{array}{c} \left[\begin{array}{cccc} 1 & 0.3 & 0.91 & 0.273\\ 0.3 & 1 & 0.273 & 0.91\\ 0.91 & 0.273 & 1 & 0.3\\ 0.273 & 0.91 & 0.3 & 1 \end{array}\right] \end{array}$$

**Figure 8:**
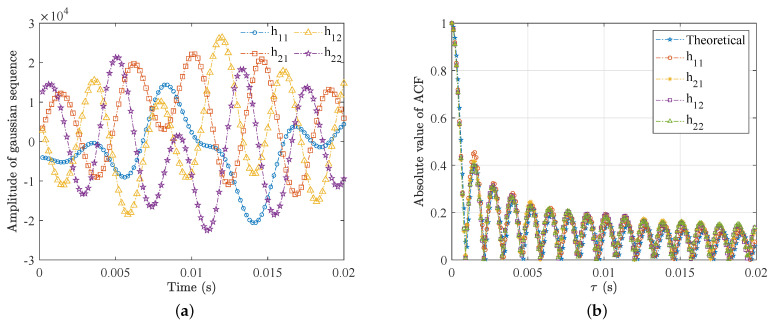
(**a**) Amplitude and (**b**) ACFs of the four Gaussian sequences.

After implementing the method of Figure 7 on an FPGA chip, we exported the output fading data using the Xilinx Chipscope tool and analyzed them using the MATLAB software. The sampling rate and period were 100 MHz and 20 ms, respectively, e.g., $2\times 10^{6}$ samples. Figure 8 depicts the amplitude of the four Gaussian sequences and the corresponding ACF. As we can see, the generated ACFs provided a quite good approximation to the theoretical ones, which can be expressed by the zero-order Bessel functions [36]. The average deviation of the ACFs between the theoretical and generated ones was about 1.26%, which can meet the wireless channel digital twin requirement.

Furthermore, the average CCF of the generated channels can be calculated as

(24) $$\begin{array}{c} \left[\begin{array}{cccc} 1.0000 & 0.2975 & 0.9101 & 0.2706\\ 0.2975 & 1.0000 & 0.2676 & 0.9097\\ 0.9101 & 0.2676 & 1.0000 & 0.2959\\ 0.2706 & 0.9097 & 0.2959 & 1.0000 \end{array}\right] \end{array}$$

which also exhibited good approximation to the desired one as (13). The mean error was merely 0.46%; the maximum error was about 1.13%; the minimum error was less than 0.01%.

## 4. Measurement Results and Validation

**Figure 9:**
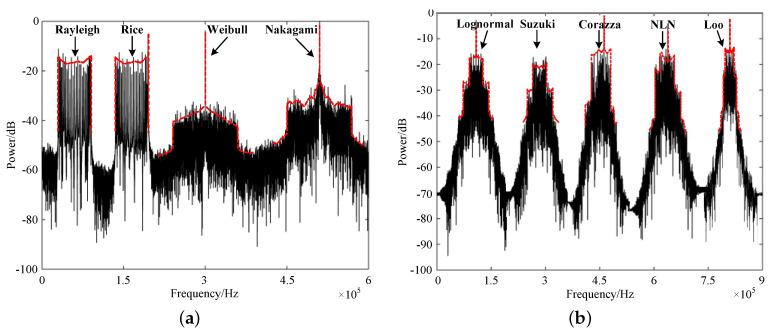
DPSD simulation results of (**a**) small-scale fading and (**b**) large-scale fading.

**Figure 10:**
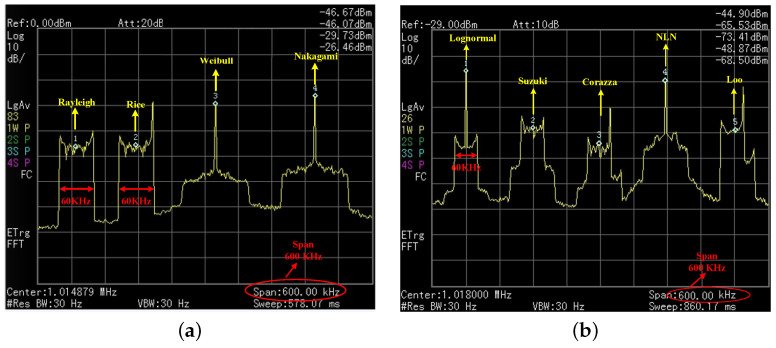
DPSD measurement results of (**a**) small-scale fading and (**b**) large-scale fading.

To validate and evaluate the proposed CORDIC-based fading generation method, we implemented the proposed arbitrary channel fading generator on a Xilinx FPGA (XC7K325TFF G900) hardware platform, where the channel consisted all the fading types in Figure 1. The system clock was 100 MHz, and the output fading data width was 16-bit. Firstly, the theoretical simulation and hardware measurement results of the DPSD of different types of channel fading were carried out as shown in Figure 9 and Figure 10. The small-scale channel fading of Rayleigh, Rice, Weibull, and Nakagami, the large-scale channel fading, and their derived fading groups, lognormal, Suzuki, Corazza, NLN, and Loo, were observed. The output of the channel emulator was divided into two groups to set four and five diameters, respectively, among which the Doppler spread of each multipath was set at 60 KHz. The measured spectrum shapes were consistent with those of the theoretical ones, which verified the correctness of the developed channel fading generator [37].

Further, Table 2 summarizes the hardware consumption in terms of the slice LUT, register, block RAM, and DSP. For comparison purposes, the hardware iteration latency is also given in Table 2 compared with the previous methods in [14,20,25,35]. As we can see, the CORDIC method had an obvious advantage with respect to the RAM consumption over the LUT methods. Note that the LUT methods needed 66 additional block RAMs (36 Kb each) to implement the trigonometric, exponential, and natural logarithm operations. By the contrast, the CORDIC-based methods needed no RAM for these operations and only consumed 18.5 block RAMs for the path delay emulation and parameter storage, i.e., the frequency, phase of each ray, and line-of sight path.

The iteration latency can be estimated by $k\times l\times \left(T_{\mathrm{Add}}+T_{reg}+T_{\mathrm{shift}}\right)\approx kT_{\mathrm{clk}}$, where *k* is the number of iterations, $T_{\mathrm{Add}}$, $T_{reg}$, and $T_{\mathrm{shift}}$ denote the delay for the l-bit carry look-ahead adder, register, and shift of the control signal in the pipeline structure, respectively, and $T_{\mathrm{clk}}$ is the basic delay of the iteration unit. In other words, the latency approximately depends on the average iteration number. As we can see, our upgraded CORDIC method reduced the iteration number to six [14,25].

**Figure 11:**
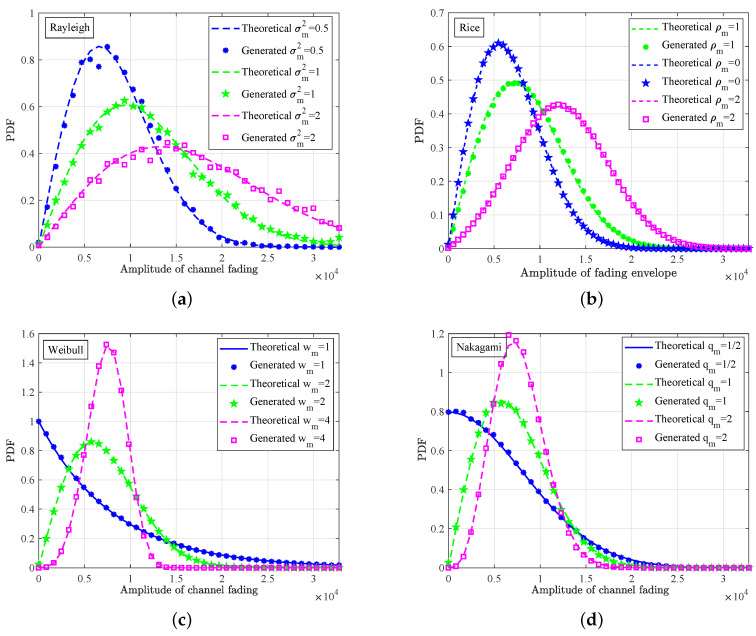
PDFs of (**a**) Rayleigh, (**b**) Rice, (**c**) Weibull, and (**d**) Nakagami fading envelope.

According to the fluctuation rate of channel fading in the time domain, it can be divided into two kinds, i.e., small-scale fading and large-scale fading. In the following, we validated the output PDFs of the small-scale fading, i.e., Rayleigh, Rice, Nakagami, and Weibull. The sampling rate was 100 MHz; the maximum Doppler frequency $f_{m}^{\max}$ was 50 Hz with the DPSD of the Jakes shape; the emulation time was 1 ms. Figure 11 shows the PDFs of $2\times 10^{6}$ generated small-scale fading samples for fivedifferent conditions, i.e., Gauss, Rayleigh, Rice, Nakagami, and Weibull. Taking the Rayleigh fading of $\sigma_{m}^{2}=1$ as an example, the theoretical mean and variance can be calculated by $\sigma_{m}\sqrt{\pi /2}$ and $\sigma_{m}^{2}\left(2-\pi /2\right)$ with the fixed-point width of 16-bit, which were 41,067 and 4,608,258,070, respectively. By stating the fading data exported from the hardware platform, the measured fixed-point mean and variance were 40,887 and 4,522,732,683, respectively, and the relative error was about 0.44% and 1.86%, respectively. Overall, the average absolute deviation of the PDFs between the theoretical and generated results in Figure 11 was approximately 1.62%.

**Figure 12:**
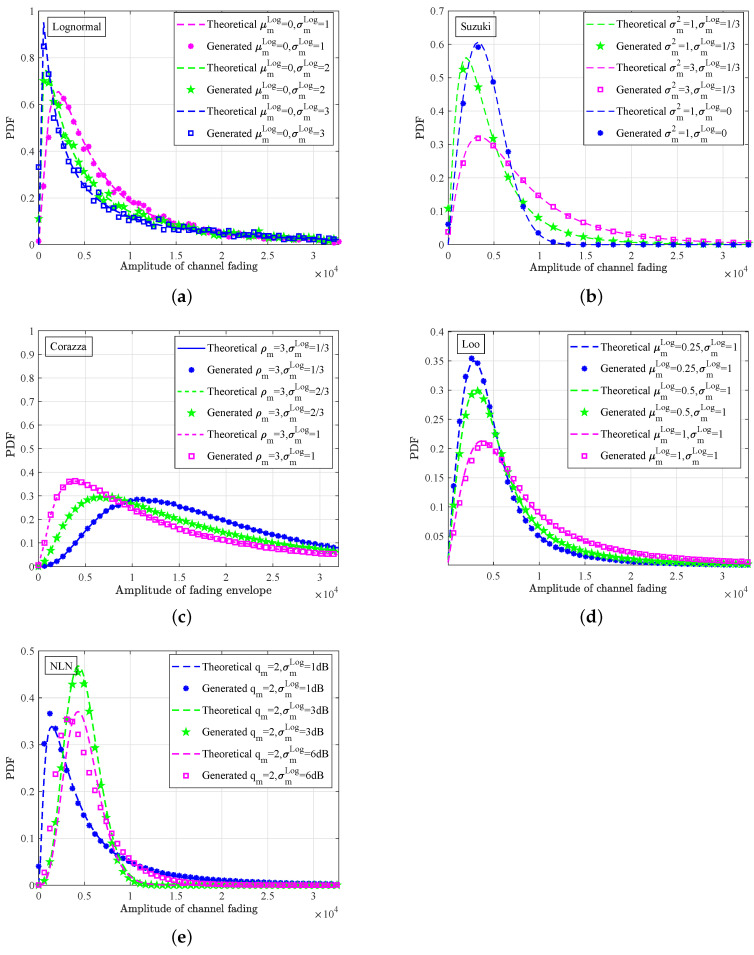
PDFs of (**a**) lognormal, (**b**) Suzuki, (**c**) Corazza, (**d**) Loo, and (**e**) NLN fading envelope.

The large-scale fading with a lognormal distribution usually occurs with different small-scale fading, which is also called composite fading [38,39], i.e., Suzuki, Loo, Corazza, and NLN. Composite fading is useful for the digital twins of terrestrial and satellite mobile channels. Figure 12 gives the PDFs of four typical composite fading types, i.e., lognormal, Suzuki, Loo, Corazza, and NLN. As we can see, the PDFs obtained from the generated $2\times 10^{6}$ channel fading samples were close to the theoretical ones. Moreover, the minimum and maximum deviations between the theoretical and generated ones were 0.0072% and 4.36%, respectively. The average deviation was about 1.63%, which proved the effectiveness and correctness of the proposed method, as well as the hardware implementation. Taking the Suzuki fading as an example, the theoretical mean and variance can be calculated by $\sigma_{m}\sqrt{\pi /2}e^{\sigma_{m}^{2}/2+\mu_{m}^{\mathrm{Log}}}$ and $\sigma_{m}^{2}e^{2\mu_{m}^{\mathrm{Log}}+\sigma_{m}^{\mathrm{Log}}}\left(2e^{\sigma_{m}^{\mathrm{Log}}}-\pi /2\right)$ with the fixed-point width of 16 bits as 48,515 and 1,828,733,997, respectively. The measured variance and mean of fixed-point hardware data were 49,213 and 1,871,364,131, and the relative error was about 2.44% and 1.44%, respectively.

## 5. Conclusions

This paper proposed an efficient generation method for multiple spatial and temporal correlated fading channels, which is also suitable for the non-stationary channel fading under time-variant scattering scenarios. With the TD-based hardware generation architecture, a greedy-CORDIC-based method was developed to perform the trigonometric, exponential, and natural logarithm operations with less RAM resource and better real-time performance than traditional methods. Moreover, the autocorrelation and cross-correlation of the output channel fading can be controlled flexibly by the users. To generate a single-channel fading on a Xilinx FPGA (XC7K325TFFG900) chip, the hardware resource was reduced from 9.14% to 6.21% over the LUT method, and the latency was cut by 62.5% over the classical CORDIC method. The hardware measurement results also demonstrated that the statistical properties of the output channel fading such as the PDF, mean, and variance were consistent with the theoretical ones. 

## Figures and Tables

**Table 1:** Different types of channel fading.

*i*	Channel Fading	Communication Scenario	References
RL	Rayleigh	Tall buildings	
R	Rice	Suburban, country	[8,10,12,13,27]
Na	Nakagami	Tall buildings, suburban, country	
Log	Lognormal	Buildings, mountains, other obstacles	
S	Suzuki	Urban	[9,14]
W	Weibull	Urban	
Loo	Loo	Country, suburban	
Co	Corazza	Highway, urban, suburban, rural	[17,18]
NLN	NLN	Mobile satellite	[19]

**Table 2:** Hardware resource consumption and latency comparison.

		Proposed	[14]	[35]	[25]
Method	LUT	CORDIC	CORDIC	CORDIC	CORDIC
Fading number	1	10	2	1	—–
System clock	100 MHz	100 MHz	100 MHz	256 MHz	—–
Slice LUTs	14,672	17,685	17,920	—–	—–
Registers	13,195	17,034	19,940		
Block RAMs	84.5	18.5	18.5		
DSPs	59	66	50		
Utilization	9.14%	6.21%	5.95%		
Average iterations	—–	5.5	16	5.5	8
Critical latency	—–	6Tclk	16Tclk	6Tclk	8Tclk

## Data Availability

Not applicable.
